# Special Issue “Disentangling Mechanisms of Genomic Regulation of Cell Functions at the Gene Level”

**DOI:** 10.3390/genes11121463

**Published:** 2020-12-07

**Authors:** Hans Binder, Arsen Arakelyan

**Affiliations:** 1 IZBI, Interdisciplinary Centre for Bioinformatics, Universität Leipzig, Härtelstr. 16–18, 04107 Leipzig, Germany; 2 Research Group of Bioinformatics, Institute of Molecular Biology NAS RA, 7 Hasratyan st, Yerevan 0014, Armenia; aarakelyan@sci.am

The term “gene” was introduced more than a hundred years ago to define a “fundamental physical and functional unit of heredity” [1]. Its use refers back to Mendel’s idea about discrete hereditable units, and it also serves as a reminiscence of Darwin’s pangenesis theory. Watson and Crick’s double helix discovery in 1962, which rationalized the molecular basis of replication and Crick’s central dogma, claiming the one-directional information flow from DNA to proteins via RNA, conceptually represent milestones linking heredity with traits, and thus, genes with cellular function. From a practical perspective, after first determination of a gene sequence (coding bacteriophage MS2 coat protein, 1972), discoveries of Sanger sequencing (1977) and polymerase chain reaction (1983) mark technological milestones that enabled the decoding of the human genome until the end of the 20th century and, with the advent of expression microarray technology, the deciphering of the human transcriptome during the following decade (ENCODE project). Instead of solving the basic mysteries of life, these discoveries probably raised more questions about gene functioning than they answered. RNA appeared as a complex information flow machinery with highly specific nucleotide interactions fulfilling a large variety of functions in fine-tuning gene activity beyond transcription and translation. Moreover, DNA appeared not only as a one-dimensional textbook of the genetic code as thought previously. Instead, it was found to represent a highly complex three-dimensional polymer, where genetic information is managed via a multitude of conformations, interactions, and molecular components. Information management includes many tasks, such as reading, writing, erasing, repairing, transferring, and translating, where each of these steps must act in a proper way in space and time to ensure the functioning of life on cellular and organismal levels. Now, the central dogma appears less dogmatic because of the multidirectional nature of these interactions including feedback loops from the transcribed and translated parts towards their information source. Biological information management involves epigenetic mechanisms forming another feedback loop linking the phenotype and environmental factors back to genetics. Presently, diverse variants of next-generation sequencing technologies that have now reached single-cell resolution together with advanced metabolomics and proteomics methods provide an immensely powerful toolbox to discover mechanisms of gene functioning on the cellular level with increasing practical impact in healthcare and biotechnology. The latest cutting-edge examples from these areas are immuno-therapy against cancer (Nobel Price in Medicine 2019), CRISPR/Cas9 gene scissor (Nobel Price in Medicine 2020), and the mRNA vaccination technique just now becoming a sword against COVID-19.

This Special Issue collects seven publications addressing different topics around genomic regulation of cell functions at the gene level as examples illustrating various aspects of this field of discovery. Two original research publications deal with temporal aspects stored in the genome on completely different timescales; one on the scale of thousands of years and the other on the scale of minutes to hours [2,3]. Both works make use of similarity relations, with one considering the genomes of vine accessions [2] and the other considering the transcriptomes of single cells extracted from the flatworm [3]. The vine genome reveals “slow” mutational modifications, which enable reconstructing paths of distribution of wine agriculture and usage from the Middle East towards Western Europe on a long time-scale over many centuries. The single-cell transcriptome of the flatworm, on the other hand, reflects relatively “fast” changes of cellular programs upon differentiation of tissues proceeding on a much shorter time scale. Both applications illustrate the impact of another methodical ingredient, namely bioinformatics (also called computational biology), in order to process huge amounts of data generated by the novel high-throughput technologies and to “translate” them into useful (systems) biological information. Algorithmic developments, data science, and computational pipelines for effective practical use are inevitable parts of realizing “from-gene-to-cell-function” discoveries. Due to the size, and most importantly, the complex, often unknown intrinsic relations between the data, machine learning is an adequate approach to extract hidden information from the data. Both papers apply self-organizing map machine learning as a so-called “molecular portrayal” approach because data are reduced into handy dimensions and visualized in terms of easy-to-interpret images on an “individual” basis, e.g., for each measured unit (vine accession or worm single cell, respectively), making it an interesting approach for personalized medicine as well.

A topic with a medical impact has been presented in the paper of Filipenko et al. [4], who reported that the protective properties of a peptide drug (Semax) against ischemic stroke are associated with the compensation of mRNA expression patterns that are disrupted during ischaemic conditions. Leitao et al. [5] reviewed the geographic distribution of genetic variants of the CYP2D6 gene which are associated with different metabolization profiles, with a focus on Amerindian populations. This study underlines the impact of genomic variability on lifestyle factors and disease incidence in different ethnicities. In their in silico study, Chetta et al. [6] addressed mechanisms of the small noncoding RNA level of genomic regulation, a field that has risen in importance only in the last 15 years. Short sequence motifs of micro- and pi-RNA transcribed from non- (protein) coding regions of the DNA modify activity via binding to mRNA and transposons, and in this way, form intricate molecular networks between the different RNA-species and transcription factors with high impact for genomic regulation. Khokhlova et al. [7] reviewed DNA repair mechanisms in the early stages of mammalian embryonic development. In general, DNA repair links cell activity back to the genome because improper function causes errors in the genetic code to accumulate and result in the appearance of diseases and/or genetic drifts. Mutations that occur in somatic cells lead to dysfunction in certain tissues or organs, while a violation of genomic integrity during the embryonic period often leads to death. A mammalian embryo’s ability to respond to damaged DNA and repair it, as well as its sensitivity to specific lesions, is not well understood. In their review, Lesne et al. [8] addressed a supramolecular aspect of genomic regulation, particularly the formation of nuclear bodies, membraneless organelles with crucial impact in regulating genome functions by promoting efficient interactions between distant genomic regions of the same or different chromosomes.

Overall, this collection of four original research papers and three reviews covers a series of mechanisms of genomic regulation and bioinformatics methods for their analysis; it provides examples and applications ranging from biotechnology to developmental biology to healthcare which will be of interest to researchers in different fields of molecular biology and medicine, agriculture, and computational biology.

## References

[1] Noble D. (2008). Genes and causation. Philos. Trans. R. Soc. A Math. Phys. Eng. Sci..

[2] Nikoghosyan M., Schmidt M., Margaryan K., Loeffler-Wirth H., Arakelyan A., Binder H. (2020). SOMmelier—Intuitive Visualization of the Topology of Grapevine Genome Landscapes Using Artificial Neural Networks. Genes.

[3] Schmidt M., Loeffler-Wirth H., Binder H. (2020). Developmental scRNAseq Trajectories in Gene- and Cell-State Space—The Flatworm Example. Genes.

[4] Filippenkov I.B., Stavchansky V.V., Denisova A.E., Yuzhakov V.V., Sevan’kaeva L.E., Sudarkina O.Y., Dmitrieva V.G., Gubsky L.V., Myasoedov N.F., Limborska S.A. (2020). Novel Insights into the Protective Properties of ACTH(4-7)PGP (Semax) Peptide at the Transcriptome Level Following Cerebral Ischaemia–Reperfusion in Rats. Genes.

[5] Leitão L.P.C., Souza T.P., Rodrigues J.C.G., Fernandes M.R., Santos S., Santos N.P.C. (2020). The Metabolization Profile of the CYP2D6 Gene in Amerindian Populations: A Review. Genes.

[6] Chetta M., Di Pietro L., Bukvic N., Lattanzi W. (2020). Rising Roles of Small Noncoding RNAs in Cotranscriptional Regulation: In Silico Study of miRNA and piRNA Regulatory Network in Humans. Genes.

[7] Khokhlova E.V., Fesenko Z.S., Sopova J.V., Leonova E.I. (2020). Features of DNA Repair in the Early Stages of Mammalian Embryonic Development. Genes.

[8] Lesne A., Baudement M.-O., Rebouissou C., Forné T. (2019). Exploring Mammalian Genome within Phase-Separated Nuclear Bodies: Experimental Methods and Implications for Gene Expression. Genes.

